# Effects of cold plasma treatment time on aroma components, amino acids, and lipids in aromatic coconut water

**DOI:** 10.1016/j.fochx.2026.103973

**Published:** 2026-05-13

**Authors:** Tao Wang, Lilan Xu, Lipin Chen, Meizhen Xie, Jiamei Wang, Weimin Zhang, Wenxue Chen, Yong-Huan Yun

**Affiliations:** School of Food Science and Engineering, Hainan University, Haikou 570228, PR China

**Keywords:** Aromatic coconut water, Cold plasma, GC-IMS, Amino acids, Lipidomics

## Abstract

Previous studies showed that cold plasma treatment affects the quality of aromatic coconut water; however, the mechanisms underlying different treatment times remains unclear. This study investigated the effects of 0, 88, and 176 s of cold plasma treatment on the contents of reactive species, aroma compounds, amino acids, and lipids. The results demonstrated that prolonged treatment generated more reactive species, with nitrite content increasing to 417.63 ± 4.20 μmol/L. 2-Acetyl-1-pyrroline was well preserved, and the content of sweet-tasting amino acids decreased significantly. Lipidomics analysis revealed that there were 25 shared differential lipids existed in coconut water across different treatment times and that cold plasma primarily promoted the oxidation of glycerides containing 18:2 or 18:1 fatty-acids. Their degradation products can be further converted into flavor compounds such as octanal. These findings provide a theoretical basis for understanding the formation and transformation pathways of aromatic compounds in coconut water under cold plasma treatment

## Highlights

• Longer cold plasma treatment times yield higher contents of reactive species.
• 2-Acetyl-1-pyrroline can be well preserved during cold plasma treatment.
• Glycine and alanine decreased significantly with the prolonged treatment time.
• Cold plasma treatment affected glycerides containing 18:2 or 18:1 most significantly.
• Reactive species promote lipid oxidation to affect aroma compounds such as octanal.

## Graphical abstract

**Figure:**
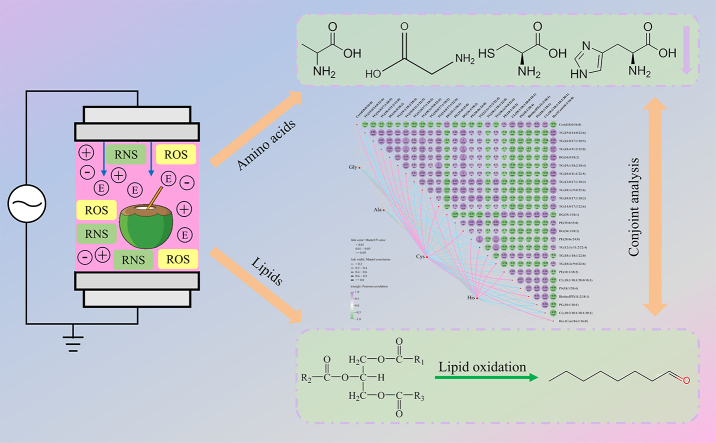

## 1 Introduction

Coconut water has a light, sweet flavor and provides diverse nutrients, including minerals, amino acids, enzymes, vitamins, and phenolic substances (Zhang et al., 2023). Aromatic coconuts are famous for their distinctive aroma and sweet taste of coconut water (Xu et al., 2025). The unique aroma of coconut water primarily originates from volatile compounds, such as esters, aldehydes, and ketones. These components not only contribute to the distinctive flavor but also possess certain physiological functions (Jermwongruttanachai et al., 2021). Owing to its high reactivity and abundance of bioactive components, coconut water is prone to oxidation and enzymatic reactions, resulting in rapid deterioration of quality (Wang et al., 2025). Accordingly, optimizing processing and storage approaches is crucial to ensure the stability and overall quality of aromatic coconut water.

Compared with conventional thermal processing, nonthermal approaches ensure product stability and microbial safety with only minimal changes to flavor and nutrient composition (Jacob et al., 2023). In recent years, cold plasma, as a nonthermal processing technology, has been widely used in fields such as food sterilization (Ekezie et al., 2017; Qiu et al., 2024), protein structure modification (Ji et al., 2018; Sharma & Singh, 2020), and food packaging (Sani et al., 2023). Cold plasma technology can generate various reactive oxygen and nitrogen species at low temperatures (Cheng et al., 2020). This process exerts significant inactivation effects on microorganisms and preserving the original physicochemical and sensory characteristics of foods (Nwabor et al., 2022). Thus, it is regarded as a potential key technology for achieving commercial sterility in food processing. Alves Filho et al. (2018) studied the chemical composition changes in American cherry juice following two sterilization methods: pasteurization and cold plasma sterilization, and the results indicated that cold plasma technology preserves the original chemical composition of the juice to the maximum extent. Kovačević et al. (2016) found that cold plasma treatment increased anthocyanin content in pomegranate juice, and the color change was clearly correlated with the treatment volume and time.

Recently, cold plasma technology has been applied to the quality control of aromatic coconut water. Current research primarily focuses on exploring the sterilizing effects of cold plasma treatment on microorganisms in aromatic coconut water, the mechanisms for maintaining key aromatic compounds, and its impact on the metabolite composition (Xu et al., 2024; Xu et al., 2025). The findings demonstrate that cold plasma technology effectively preserves the key flavor of aromatic coconut water and that cold plasma treatment significantly influences the composition and contents of lipids and amino acids. Although cold plasma exhibits notable advantages in preserving characteristic flavor and extending the shelf life of aromatic coconut water, the reactive nitrogen and oxygen species generated during the treatment process pose potential threats to its quality and safety of aromatic coconut water. Therefore, systematically investigating the concentrations of these reactive species under different cold plasma treatment times and their effects on the quality attributes of aromatic coconut water is essential.

Currently, no studies have determined the content of the generated reactive species or their impact on the quality of coconut water. Therefore, this study aimed to systematically investigate the levels of reactive species generated under different cold plasma treatment times and their effects on aroma components, amino acids, and lipids in aromatic coconut water. By quantifying reactive species generated under different cold plasma treatment times and comparing these with relevant standards, the treatment time can be optimized for subsequent applications of cold plasma technology in coconut water processing. Furthermore, by investigating the patterns of change in aroma components, amino acids, and lipids in coconut water under different cold plasma treatment times, the mechanisms by which cold plasma technology affects coconut water quality can be systematically elucidated, thereby enabling optimization of the processing methodology.

## 2 Materials and methods

### 2.1 Materials and sample preparation

The aromatic coconut was purchased from the CR Vanguard supermarket in Haikou City, Hainan Province, China. The coconut was matured for eight months. Then the coconut was shelled under aseptic conditions to collect the coconut water, and the fresh aromatic coconut water was randomly divided into five equal groups. The coconut water was treated using a DBD-type cold plasma equipment (BK 130/36, Phoenix Company, USA) with a gas composition of 49% N_2_ + 41% CO_2_ + 10% O_2_, a treatment voltage of 73 kV and a working frequency of 120 kHz (Xu et al., 2024). Five groups of coconut water were subjected to cold plasma treatment for times of 0 s, 44 s, 88 s, 132 s, and 176 s at 25 °C, with three biological replicates performed for each group.

### 2.2 Determination of pH, total plate count, and reactive species content of coconut water

The pH value, total plate count, hydrogen peroxide (H_2_O_2_) content, superoxide anion radical (O_2_^−^) content, and NO_X_ content of coconut water were measured at different cold plasma treatment times. The total plate count was determined according to the Chinese national standard GB 4789.2–2022. The contents of H_2_O_2_ and O_2_^−^ were measured using H_2_O_2_ and O_2_^−^ test kits (sulfonamide microplate method), respectively. The concentration of nitrite (NO_2_^−^) in coconut water was measured using a nitrite test kit, and the concentration of nitrate (NO_3_^−^) was indirectly measured using a nitric oxide test kit.

### 2.3 Determination of volatile compounds based on GC-IMS

Gas chromatography-ion mobility spectrometry (GC-IMS) analysis was performed using the Agilent 8890 (Agilent Technologies, USA) and IMS-Module (G.A.S, Germany). A 20-mL headspace vial with a magnetic cap was loaded with 5 mL of aromatic coconut water. Samples were incubated at 40 °C with agitation at 500 rpm for 15 min before analysis. The headspace was injected once at 500 μL, using a needle temperature of 80 °C. Each sample group underwent triplicate parallel measurements. The detection conditions for GC and IMS were referenced to Xu et al. (2024), with specific conditions listed in the supplementary material.

### 2.4 Determination of amino acid composition

The amino acid composition of coconut water was determined using the automatic amino acid analyzer method specified in GB 5009.124–2016. The aromatic coconut water samples treated with cold plasma for 0 s, 88 s, and 176 s were selected for the analysis of amino acid content, which was measured using an LA 8080 (Hitachi, Japan) amino acid automatic analyzer. A sulfonic acid cation-exchange column (4.6 mm × 60 mm × 3 μm) was used in the amino acid automatic analyzer. The column temperature was maintained at 135 ± 1 °C, and pump pressure and flow rate were maintained within 0–20 kPa and 0–0.999 mL/min, respectively. Each injection involved 20 μL of the sample. Amino acids were detected using a dual-wavelength method: proline at 440 nm and the remaining amino acids at 570 nm.

The qualification of amino acids in aromatic coconut water was based on the chromatograms and retention time parameters of amino acid standards. Amino acid levels in aromatic coconut water (mL/100 mL) were quantified using the external standard method and corresponding standard curves. The calculation method is shown in Eq. (1).

(1) $$X=\frac{c\times M\times F\times V\times 100}{20\times 10^{3}\times V_{0}}$$

Where X represents the concentration of amino acids in the sample (mg/100 mL), and C corresponds to the quantity of amino acids in a 20 μL aliquot of the test solution measured in nmol. V and V₀ indicate the final volume after hydrolysis (mL) and the sample volume used for measurement (mL), respectively. M represents the molecular weight of the amino acids, and F denotes the dilution factor.

### 2.5 Determination of lipids by UHPLC-Q-Exactive/MS

For extraction, 200 μL of aromatic coconut water was combined with 80 μL of methanol and 400 μL of methyl tert-butyl ether (MTBE), and vortexed for 30 s. Ultrasonication was performed at 5 °C and 40 kHz for 30 min, followed by centrifugation at 13,000 *g* for 15 min at 4 °C. A total of 350 μL of the resulting supernatant was evaporated to dryness and redissolved in 100 μL of an isopropanol–acetonitrile mixture (1,1, *v*/v). The reconstituted samples were vortexed for 30 s, sonicated in an ice-water bath at 40 kHz for 5 min, and then centrifuged at 13,000 *g* for 5 min at 4 °C. Finally, 80 μL of the supernatant was transferred to a 2-mL autosampler vial containing a glass insert for subsequent analysis.

Aromatic coconut water was analyzed using a UHPLC-Q Exactive HF-X system (Thermo Fisher Scientific), with the method referenced from the research of Zhang, Li, et al. (2025) and appropriately modified. Chromatographic separation was performed on an Accucore C30 column (100 mm × 2.1 mm × 2.6 μm), injecting 2 μL of the samples per run. The mobile phases were prepared as follows: phase A, 10 mM ammonium acetate in 50% acetonitrile-water with 0.1% formic acid; phase B, 12 mM ammonium acetate in acetonitrile-isopropanol-water (10,88,2, *v*/v/v) containing 0.02% formic acid. The column temperature was maintained at 40 °C. The flow rate was constant at 0.40 mL/min throughout the entire separation process. The mobile phase separation gradient was as follows: the composition of the mobile phase started with 65% solvent A and 35% solvent B at time 0 min. At 4 min, the composition was adjusted to 40% solvent A and 60% solvent B. At 12 min, solvent A content was decreased to 15%, and solvent B content was increased to 85%. Between 15 and 17 min, the mobile phase was 100% solvent B. From 18 min onward, the mobile phase was again 65% solvent A and 35% solvent B, continuing until 20 min.

MS data were collected in positive and negative ion scanning modes, covering an *m*/*z* range from 200 to 2000. The ion spray voltage was adjusted to +3000 V for positive ions and −3000 V for negative ions. The electrospray source temperature was maintained at 370 °C, and the sheath and auxiliary gases were maintained at 60 psi and 20 psi, respectively. Ion fragmentation was conducted using a stepped collision energy protocol of 20, 40, and 60 V.

### 2.6 Data processing

Significance tests were performed via one-way analysis of variance (one-way ANOVA) and post-hoc Tukey's test, and differences were considered significant when *p* < 0.05. The volatile compound fingerprints were obtained via the Gallery Plot tool integrated in the VOCal software of the GC-IMS system. The statistical differences were analyzed using SPSS 26 (IBM, USA), while data visualization and multivariate analysis were performed using Origin 2022 (OriginLab, USA), GraphPad Prism 8 (GraphPad Software, USA), MetaboAnalyst 6.0 (https://www.metaboanalyst.ca/), and the Majorbio Cloud Platform (https://cloud.majorbio.com/).

## 3 Results and discussion

### 3.1 Analysis of the pH and active species content of coconut water with different treatment times

**Fig. 1:**
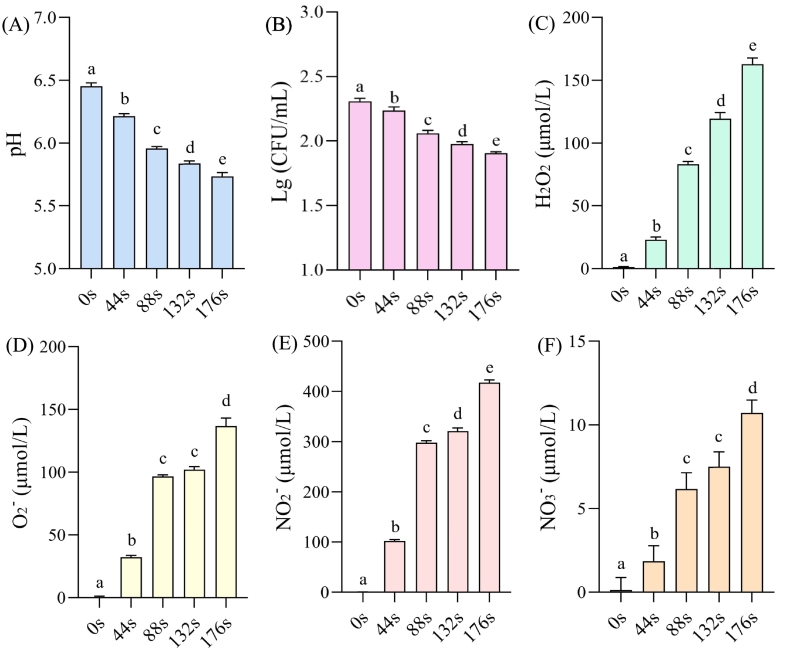
The pH (A), total plate count (B), H_2_O_2_ (C), O_2_^−^ (D), NO_2_^−^ (E), and NO_3_^−^ (F) of aromatic coconut water samples with different cold plasma treatment times.

Fig. 1A illustrates that the pH of aromatic coconut water decreased with the increasing treatment time. A significant pH decrease was observed in the treated samples compared with the untreated one, which may result from acidic species (such as H^+^, nitrous acid, and nitric acid) formed by reactions between water and high-energy reactive species generated via cold plasma ionization of air. As shown in Fig. 1B, the total plate count in aromatic coconut water gradually decreased with increasing cold plasma treatment time.

Various reactive species, including free radicals, positive and negative ions, electrons, and molecules, are generated during the discharge of cold plasma. The shielding and protective nature of liquids limits the direct action of these plasma-derived species on bacteria in liquid systems (Machala et al., 2013). They first interact with the liquid through a series of physicochemical processes, forming reactive nitrogen and oxygen species that subsequently interact with the bacteria to achieve sterilization (Jamróz et al., 2014). H_2_O_2_ and O_2_^−^ are the predominant reactive oxygen species (ROS) generated by plasma. H_2_O_2_ is primarily formed via the recombination of ·OH generated by electrical discharges. O_2_^−^ is mainly produced through the excitation or ionization of O_2_ induced by collisions with high-energy electrons. The changes in the concentrations of H_2_O_2_ and O_2_^−^ observed in this study are shown in Fig. 1C and Fig. 1D. Increasing the plasma treatment time resulted in a steady increase in the concentrations of H_2_O_2_ and O_2_^−^ in aromatic coconut water. When the treatment time was extended from 0 s to 176 s, the contents of H_2_O_2_ and O_2_^−^ increased from 1.17 ± 0.28 μmol/L and 0.58 ± 0.52 μmol/L to 162.75 ± 4.03 μmol/L and 136.81 ± 5.18 μmol/L, respectively.

Some high-energy reactive species generated by cold plasma are also signaling molecules in cells. At low concentrations, they play essential biological roles. However, their high reactivity leads to interactions with the cell membrane and intracellular structures when their levels increase (Rhee, 2006). Taking H_2_O_2_ as an example, it can participate in intracellular signal transduction processes when its concentration is relatively low. However, at higher concentrations, H_2_O_2_ may cause damage to lipids, proteins, and DNA in cells (Ransy et al., 2020). Therefore, during plasma sterilization, careful control of treatment time is necessary to balance microbial inactivation and the preservation of coconut water quality by maintaining appropriate concentrations of reactive substances such as H_2_O_2_. In addition, according to Misra et al. (2016), the ROS such as O_2_^−^ generated by cold plasma treatment can induce chemical alterations in the side chains of protein amino acid residues. These alterations lead to changes in the side-chain structure and a reduction in α-helical content, ultimately resulting in decreased or complete loss of enzyme activity. Therefore, prolonged treatment may further inhibit the activity of polyphenol oxidase in aromatic coconut water, which in turn may reduce browning to some extent.

In addition to ROS, cold plasma treatment generates a substantial amount of reactive nitrogen species (RNS), which predominantly exist as nitrates and nitrites under acidic conditions. The formation of RNS is predominantly attributed to electron collisions with N_2_ present in the working gas, which generate N and NO, which are subsequently further oxidized to NO_2_. As shown in Fig. 1E and Fig. 1F, as the treatment time increased, aromatic coconut water showed a significant increase (*p* < 0.05) in content of reactive species such as NO_2_^−^ and NO_3_^−^, consistent with the pH trends. The concentration of nitrite remarkably increased from 0.31 ± 0.18 μmol/L to 417.63 ± 4.20 μmol/L. Notably, the nitrite content in the aromatic coconut water after 88 s of cold plasma treatment exceeded the maximum limit of 20 mg/kg specified in GB 2762–2022, which may affect the safety of foods. The increased nitrite concentrations in foods can severely compromise safety and pose substantial threats to human health, potentially resulting in poisoning, cancer development, or even death (Karwowska & Kononiuk, 2020).

O_2_^−^ is generated when high-energy electrons collide with O_2_, resulting in the capture of an electron by O_2_ to form O_2_^−^. High-energy electrons dissociate N_2_ and O_2_ in the air, and the resulting N and O atoms recombine to produce NO. NO further reacts with O_3_ or other oxides in the gas phase to form NO_2_. NO and NO_2_ dissolve in the aqueous solution and react with water, yielding NO_2_^−^ and NO_3_^−^. In summary, the increase in contents of reactive nitrogen and oxygen species during cold plasma treatment is correlated with the proportion and concentration of N_2_ and O_2_ in the gas composition. Therefore, in subsequent research, altering the gas used in cold plasma processing, such as substituting nitrogen with argon, could be explored as a means to minimize the generation of nitrites in aromatic coconut water and improve its safety profile.

### 3.2 Effect of different treatment times on the aroma components of coconut water

Aromatic coconut water's quality is largely determined by its aroma, which originates from various volatile compounds. Variations in both the composition and concentration of these volatiles significantly affect flavor perception, resulting in distinct aroma profiles (Du et al., 2025). GC-IMS was used to profile the volatile compounds in aromatic coconut water and reveal their dynamic changes during varying cold plasma treatment times. Detected volatiles were qualitatively analyzed by comparing their retention indices and drift times with corresponding values obtained from reference standards. A total of 31 volatile aroma compounds (corresponding to 37 peak signals), including monomers and several dimers, were identified in coconut water. These compounds were classified into six categories: 13 esters, 5 alcohols, 5 aldehydes, 2 ketones, 4 heterocyclic compounds, and 2 other compounds. The specific information of each compound is listed in Table S1.

**Fig. 2:**
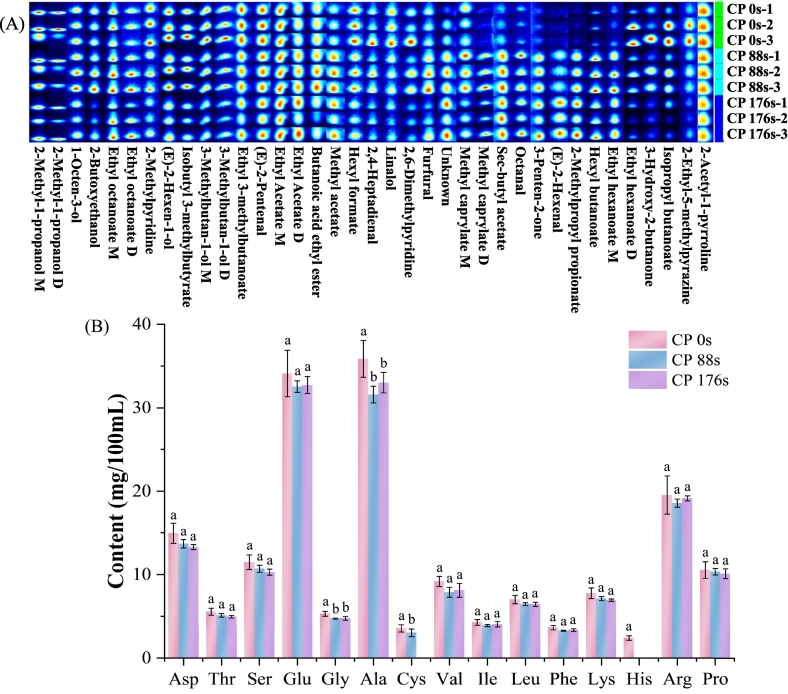
The fingerprints of volatile components (A) and content of amino acids (B) in coconut water with different cold plasma treatment times.

Fig. 2A illustrates the GC-IMS fingerprints of aromatic coconut water after various cold plasma treatment times. During ionization, highly concentrated volatile organic compounds may form monomers, dimers, or trimers, resulting in multiple signal peaks originating from a single substance (Yao et al., 2024). In this study, both monomeric and dimeric forms of isobutanol, isoamyl alcohol, ethyl acetate, methyl octanoate, ethyl octanoate, and ethyl hexanoate were identified, indicating that all six compounds were present at relatively high concentrations. Given the low odor thresholds of esters, these compounds play a crucial role in generating sweet and fruity aromas, typically formed through esterification reactions between acids and alcohols. Among the volatile compounds, esters were the most numerous and had the highest concentrations, contributing substantially to its pleasant flavor profile.

Alcohols are largely generated via lactose and amino acid metabolism, imparting characteristic alcoholic, fruity, and sweet scents (Nie et al., 2023). In this study, alcohols such as isobutanol and isoamyl alcohol were identified, which were characterized by alcoholic, malty, banana, and fruity notes, thereby enhancing the overall flavor of coconut water. Alcohols may also be generated through the oxidative degradation of lipids (Zhao et al., 2025). 1-Octen-3-ol is predominantly produced through the thermal oxidative breakdown of arachidonic acid, causing rise to its distinctive mushroom-like scent (Sohail et al., 2022; Xu et al., 2023). In general, unsaturated alcohols have a stronger impact on flavor than their saturated counterparts, largely owing to their lower sensory detection thresholds (Wang et al., 2022). In this study, 1-octen-3-ol, trans-2-hexen-1-ol, and linalool were classified as unsaturated alcohols, whereas isobutanol and isoamyl alcohol were not.

Aldehydes, formed through fatty acid metabolism and amino acid breakdown, represent essential flavor constituents in various foods due to their extremely low odor thresholds (Zhu et al., 2021). Arshad et al. (2018) indicated that linear aldehydes are largely derived from lipid oxidation, while branched aldehydes mainly come from the Strecker degradation of amino acids. In this study, the aldehydes were predominantly linear-chain types. Therefore, the aldehydes in aromatic coconut water are mainly derived from the oxidative degradation of lipids.

Fig. 2A illustrates that the concentrations of compounds such as hexyl formate, linalool, and isobutyl butyrate markedly decreased with the prolonged cold plasma treatment, indicating that excessive treatment time may result in the loss of the sweet and fruity aroma characteristics of aromatic coconut water. The concentrations of aldehydes and ketones such as octanal, (E)-2-hexenal, and 3-penten-2-one showed an increased trend as the cold plasma treatment time was extended. This indicates that cold plasma treatment can promote the lipid oxidation process in aromatic coconut water, thereby altering its flavor characteristics. In addition, 2-acetyl-1-pyrroline, which is characterized by a sweet and popcorn aroma, is a key characteristic volatile compound in aromatic coconut water. The concentration of 2-acetyl-1-pyrroline decreased as the cold plasma treatment time increased, but the change was relatively small, indicating that the characteristic aroma of coconut water was well preserved. Notably, octanal, a key volatile compound contributing to the aroma of plasma-treated coconut water (Xu et al., 2025), markedly increased with the prolonged treatment time. This effect is likely due to lipid oxidation facilitated by the reactive oxygen and nitrogen species generated throughout the cold plasma treatment process.

### 3.3 Effect of different treatment times on the composition of amino acids in coconut water

Free amino acids are not only the primary constituents of proteins but also key contributors to the taste of various foods. Moreover, they serve as essential precursors of flavor compounds. Free amino acid profiles and their levels in foods are widely recognized as key indicators for assessing product quality and sensory characteristics (Xiang et al., 2023). To investigate how varying times of cold plasma treatment influence the amino acid profile and levels in aromatic coconut water, the free amino acid contents of the samples treated for 0, 88, and 176 s were determined. The results are presented in Fig. 2B and Table S2, and a total of 15 amino acids were identified across the three groups of coconut water samples.

The predominant free amino acids in aromatic coconut water were alanine and glutamic acid, with concentrations of 35.86 ± 2.19 and 34.11 ± 2.78 mg/100 mL in the CP_0s group, respectively. Conversely, cysteine and histidine were present at relatively low levels. Based on taste properties, free amino acids are categorized into three main types: umami, sweet, and bitter. Among them, glutamic acid is an umami amino acid, whereas alanine is a sweet amino acid. The aromatic coconut water primarily contains umami and sweet amino acids, which play a crucial role in its desirable flavor profile.

The exposure of aromatic coconut water to cold plasma for 88 and 176 s showed a significant decrease in the content of total free amino acid content compared with untreated samples, particularly glycine and alanine (*p* < 0.05). The reduction in content of these sweet-tasting amino acids could reduce the overall sweetness of coconut water. Misra et al. (2016) reported that reactive species produced by plasma sources may induce chemical modifications in amino acid side chains. Moreover, cold plasma treatment triggers the Maillard reaction, which consumes specific free amino acids (Yu et al., 2020). Given that alanine and glycine serve as key substrates for the Maillard reaction, these factors may collectively contribute to the reduction in free amino acid content.

As shown in the Fig. 2B and Table S2, cysteine was not detected in aromatic coconut water after 88 s of cold plasma treatment, and both cysteine and histidine disappeared after 176 s of treatment. This may be attributed to the direct oxidation of readily oxidizable side chain groups, such as the –SH groups of cysteine and the imidazole groups of histidine, by potent oxidizing species, including reactive nitrogen and oxygen species generated during cold plasma treatment, ultimately resulting in structural disruption or conversion into other products. Moreover, the longer the treatment time was, the more pronounced was the effect on amino acids. Yan et al. (2015) revealed that the reactive species generated by cold plasma treatment primarily react with cysteine, which is consistent with the results of this study.

In addition, six essential amino acids, namely, threonine, phenylalanine, leucine, isoleucine, valine, and lysine, were detected in aromatic coconut water. The aromatic coconut water showed a relatively high content of essential amino acids, with a concentration of 37.41 ± 2.83 mg/100 mL in the CP_0s group. The concentrations of these amino acids showed no significant variation during cold plasma treatment, implying that the treatment did not disrupt amino acid stability and preserved the essential nutrients beneficial to human health.

### 3.4 Effect of different treatment times on the lipid composition of coconut water

#### 3.4.1 Lipid composition of aromatic coconut water

Lipids, characterized by specific nutritional and bioactive functions, are key energy sources and essential precursors for the formation of volatile flavor compounds in plants (Zhang, Hua, et al., 2025). Lipids are key food constituents that not only deliver essential fatty-acids but also modulate flavor evolution and off-flavor development via oxidation and degradation pathways. A total of 423 lipid metabolites were identified in this study.

**Fig. 3:**
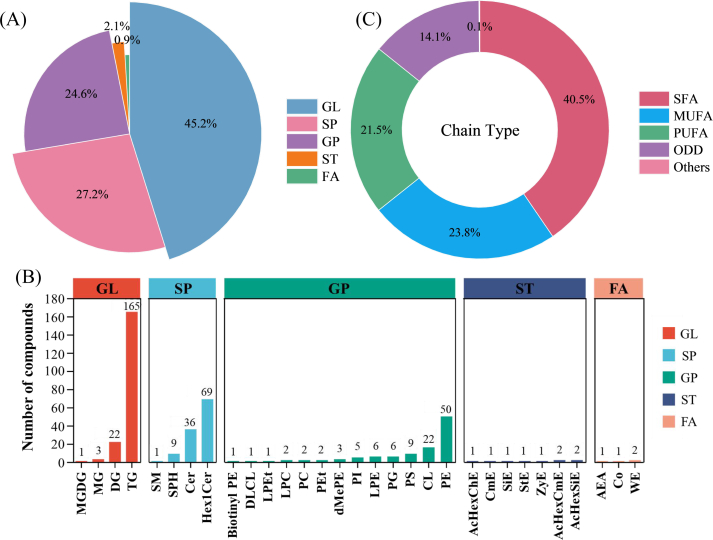
The lipid composition in coconut water under different classification criteria. (A) classes; (B) subclasses; (C) degree of unsaturation.

Based on lipid metabolic pathways, lipids can be classified into 8 primary categories: glycerophospholipids (GP), glycerolipids (GL), fatty acyls (FA), sphingolipids (SP), saccharolipids (SL), sterol lipids (ST), prenol lipids (PR), and polyketides (PK), which can be further subdivided into 96 distinct subclasses. Fig. 3A and Fig. 3B illustrate that aromatic coconut water contains 423 lipid metabolites, which are distributed among 5 major lipid classes: 191 GL, 104 GP, 115 SP, 9 ST, and 4 FA. These lipids can be further divided into 31 subclasses. GL mainly includes 165 triglycerides (TG), 22 diglycerides (DG), 3 monoglycerides (MG) and 1 monogalactodiglyceride (MGDG). SP comprises 69 hexosylceramides (Hex1Cer), 36 ceramides (Cer), 9 sphingosines (SPH) and 1 sphingomyelin (SM). GP primarily comprises 50 phosphatidylethanolamines (PE), 22 cardiolipins (CL), 9 phosphatidylserines (PS), 6 lysophosphatidylethanolamines (LPE), 6 phosphatidylglycerols (PG), 5 phosphatidylinositol (PI), 3 dimethylphosphatidylethanolamines (dMePE), 2 phosphatidylethanols (PEt), 2 phosphatidylcholines (PC), 2 lyso-phosphatidylcholines (LPC), 1 lyso-phosphatidylethanol (LPEt), 1 dilinoleoyl cardiolipin (DLCL) and 1 biotinylated phosphatidylethanolamine (Biotinyl PE). ST comprises 2 acetylated hexose-substituted sitosterol ester (AcHexSiE), 2 acetylated hexose-substituted campesterol ester (AcHexCmE), 1 zymosteryl (ZyE), 1 stigmasteryl ester (StE), 1 sitosterol ester (SiE), 1 campesterol ester (CmE) and 1 acetylated hexose-substituted cholesterol ester (AcHexChE). FA primarily consists of 2 wax ester (WE), 1 coenzyme (Co) and 1 arachidonylethanolamide (AEA). The results indicated that the lipids in aromatic coconut water were predominantly composed of GL, SP, and GP, accounting for approximately 97% of the total lipid content. Among these subclasses, triglycerides were the most abundant lipid species in aromatic coconut water.

Moreover, lipid metabolites can also be grouped according to their unsaturation level into saturated fatty acyls (SFAs), monounsaturated fatty acyls (MUFAs), polyunsaturated fatty acyls (PUFAs), and odd-chain fatty acyls (ODDs). As shown in Fig. 3C, lipids in aromatic coconut water were predominantly composed of SFAs, followed by MUFAs and PUFAs.

#### 3.4.2 Multivariate statistical analysis

**Fig. 4:**
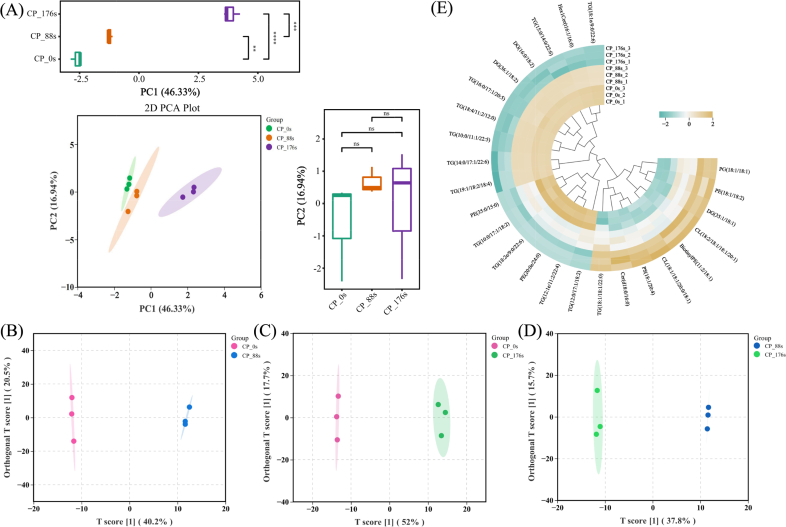
The PCA score plot (A), OPLS-DA score plots (B-D) and the clustering heatmap of differential lipids (E) of coconut water with different cold plasma treatment times.

Principal components analysis (PCA) and orthogonal partial least squares discriminant analysis (OPLS-DA) were then used to visualize the overall variation among samples and to identify treatment-dependent metabolic changes. As an unsupervised technique, PCA is frequently applied to depict sample distributions according to their inherent relationships (Cai et al., 2021). Based on lipid peak intensity data, the PCA model effectively separated aromatic coconut water samples into three distinct groups (Fig. 4A). As shown in the PCA score plot, a clear distinction existed between fresh aromatic coconut water and that treated with cold plasma, highlighting the significant impact of plasma treatment on lipid composition of coconut water. Moreover, a progressive shift along the PC1 axis was observed with the increasing treatment time, indicating a consistent influence of treatment time on the lipids in aromatic coconut water. The bar chart indicates that the differences among samples with different treatment times were primarily observed in PC1, with no significant differences in PC2. More extensive changes in lipid compounds were observed as the cold plasma treatment time increased.

As shown in Figs. 4B–D, supervised OPLS-DA models were constructed to discriminate among the three comparison groups: CP_0s vs. CP_88s, CP_0s vs. CP_176s, and CP_88s vs. CP_176s. Cold plasma processing markedly altered the lipid profile of coconut water, and these alterations were effectively discriminated using OPLS-DA. The R^2^ values of the CP_0s vs. CP_88s, CP_0s vs. CP_176s, and CP_88s vs. CP_176s models were 0.974, 0.982, and 0.98, respectively. The Q^2^ values were 0.82, 0.916, and 0.8, respectively. The R^2^ and Q^2^ values were both close to 1, indicating that the predictive and explanatory performance of the models for the two groups was excellent. Compared with the PCA score plot, OPLS-DA demonstrated a higher degree of separation and discrimination. Consistent with the results of the PCA, the OPLS-DA demonstrated that longer cold plasma treatments progressively altered the lipid composition in aromatic coconut water.

#### 3.4.3 Differential lipid analysis

Given the high-dimensional and large-scale nature of omics datasets, an integrative analytical framework combining univariate and multivariate statistical approaches is essential for achieving a multidimensional understanding. This integration allows the accurate discrimination of differential metabolites and reveals the temporal dynamics of lipid metabolism in aromatic coconut water subjected to cold plasma treatments. The variable importance in projection (VIP) values derived from multivariate statistical analysis using OPLS-DA can be used for the preliminary screening of differential metabolites between groups. By integrating these results with the *p*-values obtained from univariate analysis, a more reliable strategy for identifying significant differential metabolites can be established. The differential lipids between the two groups were determined according to the thresholds of *p* < 0.05 and VIP ≥ 1, and the top 30 differential lipids between each of the two groups are presented in Tables S3–S5.

A total of 174 differential lipids were identified across the three comparison groups, with 25 lipids being the shared differential lipids. To provide a visual representation of their changing patterns, these 25 lipids were displayed in a heatmap, as depicted in Fig. 4E. As depicted in Fig. 4E, prolonged cold plasma processing resulted in a gradual decrease in content in the majority of the differential lipids, such as TG (15:0/14:0/22:6), TG (16:0/17:1/20:5), TG (18:4/11:2/12:0), TG (19:1/18:2/18:4), and TG (12:0/17:1/18:2). Conversely, the contents of a small number of lipids, such as Cer (d18:0/16:0), PG (18:1/18:1), PE (18:1/18:2), PS (18,1/20:4) and CL (18,2/18:1/18:1/20:1), increased trend as the treatment time increased. Notably, most of these differential lipids are PUFAs. PUFAs are prone to oxidative breakdown, and the products formed during this process have a direct impact on volatile flavor composition (Storrustløkken et al., 2015). This study demonstrated that triglycerides and diglycerides might undergo oxidative degradation during cold plasma treatment, which aligns with the observations reported by Wang et al. (2024). Moderate lipid oxidation promotes the development of characteristic aroma molecules (Zhai et al., 2024). In this study, prolonged cold plasma treatment decreased the contents of both total lipids and differential lipids in aromatic coconut water. Considering the temporal changes in O_2_^−^, NO_2_^−^, and NO_3_^−^ generated during plasma treatment, cold plasma treatment is speculated to promote the oxidative degradation of lipids by producing more reactive oxygen and nitrogen species, thereby generating a higher number of volatile compounds such as octanal, (E)-2-hexenal, and 3-penten-2-one. The process intensifies the aroma in aromatic coconut water, consistent with the changes in volatile compounds across different treatment times.

**Fig. 5:**
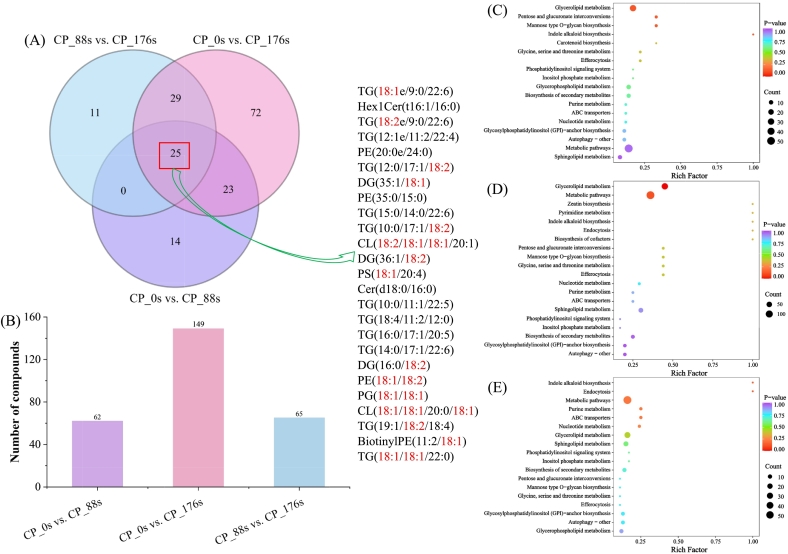
The analysis of differential lipids in coconut water under different treatment times. (A) Venn diagram and (B) histogram; (C–E) KEGG enrichment results for the comparisons of CP_0s vs. CP_88s, CP_0s vs. CP_176s, and CP_88s vs. CP_176s.

Fig. 5A and Fig. 5B illustrate that the number of differential lipids increases as the treatment time increases. The results imply that the quality of aromatic coconut water is more substantially affected by prolonged cold plasma treatment, consistent with the outcomes of PCA. In addition, these comparison groups shared 25 differential lipids. Notably, most of them were triacylglycerols and diacylglycerols, which showed a decreasing trend with prolonged treatment time. Their structures were mainly characterized by 18:2 and 18:1 acyl residues (linoleoyl and oleoyl), indicating that they are mainly unsaturated glycerides. Unsaturated glycerides act as key precursors of aroma in food processing, where they may be hydrolyzed by lipase to generate unsaturated free fatty-acids (Zhang, Hua, et al., 2025). The triglycerides and diglycerides containing an 18:2 or 18:1 fatty-acids generated linoleic acid and oleic acid through oxidative and degradation processes.

Linoleic acid and oleic acid can be further converted into aldehydes, esters, ketones, and alcohols via α-oxidation, β-oxidation, and lipoxygenase pathways, thereby influencing the flavor characteristics of fruits (Cao et al., 2020). Therefore, cold plasma may promote the degradation of glycerides in aromatic coconut water, enhancing the biosynthesis of volatile aroma compounds with fresh, floral, and fruity aromas and ultimately improving its overall aroma quality. Specifically, triglycerides and diglycerides containing of 18:2 and 18:1 fatty-acids are likely to serve as potential key precursors for the formation of these aroma compounds. Combined with variations in aroma components in aromatic coconut water determined by GC-IMS, cold plasma can be speculated to promote the oxidation and degradation of triglycerides and diglycerides containing of 18:2 and 18:1 fatty-acids, facilitating the generation of oleic acid and linoleic acid. Furthermore, these compounds may participate in the transformation reactions that yield volatile flavor compounds such as octanal.

Figs. 5C–E present the Kyoto Encyclopedia of Genes and Genomes (KEGG) enrichment analysis of differential lipids under different cold plasma treatment times. Across the three comparison groups of CP_0s vs. CP_88s, CP_0s vs. CP_176s, and CP_88s vs. CP_176s, 18, 21, and 18 metabolic pathways were enriched, respectively. Notably, the differential lipids in all comparisons were predominantly enriched in metabolic pathways and glycerolipid metabolism, suggesting that diacylglycerols, triacylglycerols, and their synthesis and degradation processes may serve as primary targets of cold plasma treatment. Collectively, these findings reveal that cold plasma treatment can modulate key lipid pathways such as glycerolipid metabolism, thereby altering the lipid composition of aromatic coconut water and consequently affecting its quality.

#### 3.4.4 Verification of flavor formation mechanisms

**Fig. 6:**
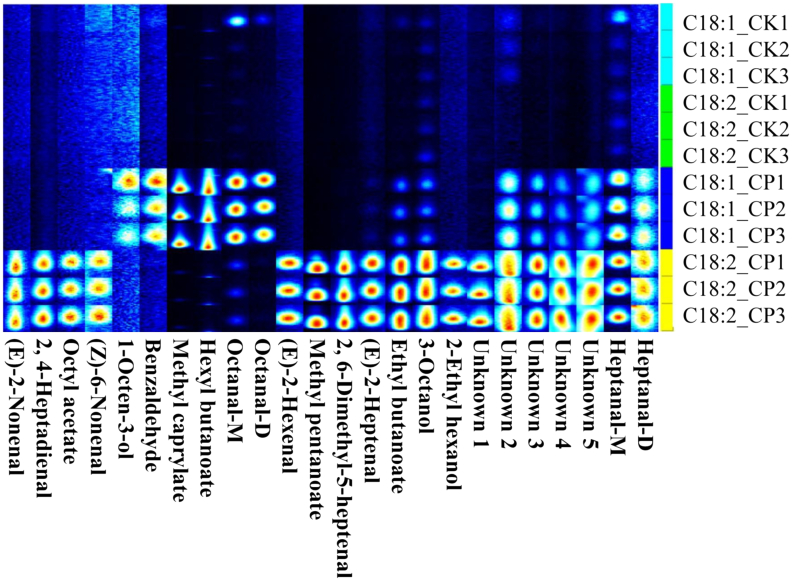
GC-IMS fingerprint profile for the verification of flavor formation mechanisms.

To further validate the effect of cold plasma treatment on the oxidation of oleic acid (C18:1) and linoleic acid (C18:2), a precursor addition experiment was conducted. Solutions of oleic acid and linoleic acid were prepared at a concentration of 1 μL/mL. The aqueous solutions were then subjected to cold plasma treatment using a cold plasma device for 88 s. The volatile compounds generated from the oleic acid and linoleic acid solutions after cold plasma treatment were analyzed using GC-IMS. The fingerprint profile of the volatile compounds in the samples is shown in Fig. 6. The CK and CP groups represent the untreated control and the cold plasma-treated groups, respectively. A total of 22 volatile compounds were detected across the four sample groups, including five unknown compounds. All of the identified volatiles were aldehydes, esters, and alcohols, with octanal and heptanal also being detected in their dimeric forms.

As illustrated in the fingerprint profile, almost no volatile compounds were detected in untreated solutions of oleic acid and linoleic acid. After treatment with cold plasma, 12 volatile compounds were generated in the oleic acid solution, including heptanal, octanal, hexyl butyrate, methyl octanoate, benzaldehyde, 1-octen-3-ol, ethyl butyrate, 3-octanol, and 4 unidentified volatile compounds. In the linoleic acid solution, cold plasma treatment resulted in the formation of a higher diversity of volatile compounds, producing a total of 18 volatile compounds: heptanal, octanal, (E)-2-nonenal, (E)-2-hexenal, (E)-2-heptenal, (Z)-6-nonenal, 2,4-heptadienal, ethyl butyrate, octyl acetate, methyl 2-methylpentanoate, 2,6-dimethyl-5-heptenal, 3-octanol, 2-ethylhexanol, and 5 unidentified compounds.

The results of the precursor addition experiments indicated that cold plasma treatment indeed exerts an oxidative effect on oleic acid and linoleic acid. Furthermore, oleic acid serves as a precursor for the following flavor compounds: heptanal, octanal, hexyl butyrate, methyl octanoate, benzaldehyde, 1-octen-3-ol, ethyl butyrate, and 3-octanol. In contrast, linoleic acid acts as a precursor for heptanal, octanal, (E)-2-nonenal, (E)-2-hexenal, (E)-2-heptenal, and (Z)-6-nonenal. Notably, octanal, which was identified as a key aroma compound in aromatic coconut water treated with cold plasma in the previous study of Xu et al. (2024), was detected in both oleic acid and linoleic acid solutions after cold plasma treatment. These results suggest that octanal is indeed likely produced from the oxidative degradation of oleic acid and linoleic acid induced by the reactive species generated by cold plasma.

### 3.5 Correlation analysis using the Mantel test

The distinctive taste of coconut water arises from the interplay of its volatile compounds, amino acids, and lipids. This study demonstrates that reactive species generated by cold plasma significantly affect lipids significantly. Lipid oxidation and degradation can alter the composition of volatile compounds and amino acids, thereby resulting in changes in the overall flavor. Then, this study focuses on elucidating correlations among significantly changed volatile compounds, amino acids, and differential lipids during cold plasma treatment.

#### 3.5.1 Mantel test analysis of aroma compounds and differential lipids

GC-IMS analysis revealed that cold plasma treatment significantly altered the volatile compound profile of coconut water significantly, with reactive species generated during treatment exerting a profound influence. Increases in aldehyde and ketone levels, particularly octanal, (E)-2-hexenal, and 3-penten-2-one, were identified as contributors to the enriched fresh flavor attributes. Consequently, Mantel test analysis was performed between these three key volatile compounds and differential lipids.

**Fig. 7:**
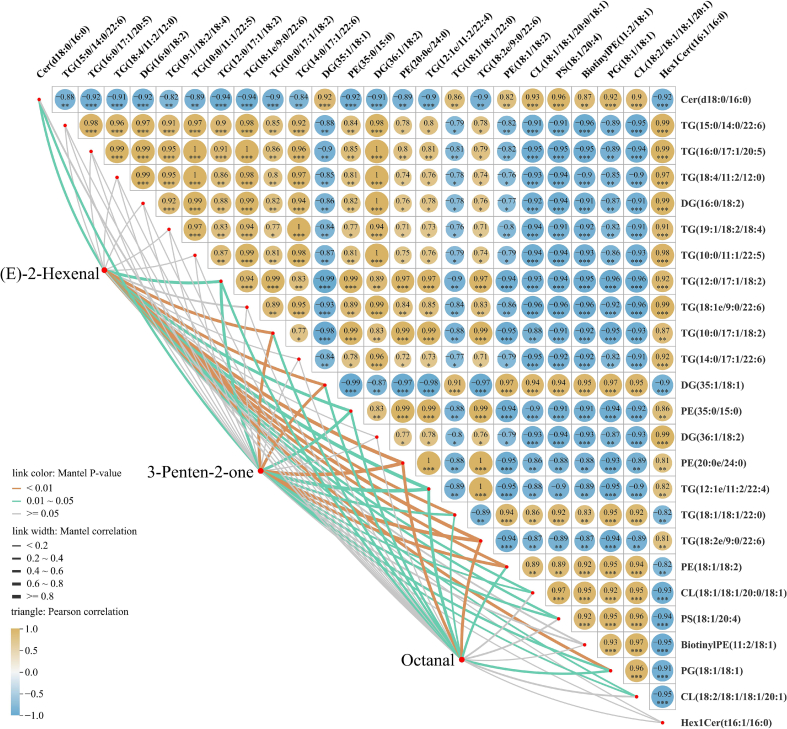
Mantel test analysis of volatile compounds and differential lipids.

The correlation analysis presented in Fig. 7 and Table S6 showed that the 25 differential lipids identified under various cold plasma treatment times were strongly interrelated, with more positive (156 pairs) than negative (144 pairs) correlations. This suggests that these lipids may be regulated by similar metabolic pathways or oxidative processes. In particular, phospholipids and glycerides containing unsaturated fatty acid residues changed synergistically, indicating that cold plasma treatment may trigger lipid peroxidation chain reactions, resulting in oxidative degradation and structural reorganization of lipids. A total of 80 pairs of lipids exhibited absolute correlation coefficients greater than 0.95. Among them, PE (20:0e/24:0) showed the strongest positive correlation with TG (12:1e/11:2/22:4) (*r* = 0.9992), whereas TG (12:0/17:1/18:2) displayed the strongest negative correlation with DG (35:1/18:1) (*r* = −0.9890).

The Mantel test results of volatile compounds and differential lipids presented in Table S7 identified 38 statistically significant correlation pairs. Among these, TG (18:2e/9:0/22:6) exhibited the strongest correlations with (E)-2-hexenal (*r* = 0.8663), 3-penten-2-one (*r* = 0.8475), and octanal (*r* = 0.6491). The findings suggest that the influence of cold plasma treatment on volatile compounds in coconut water is associated with the oxidative degradation of glycerides with 18:2 or 18:1 fatty-acids. Notably, octanal, (E)-2-hexenal, and 3-penten-2-one exhibited a higher proportion of positive correlations than negative correlations with differential lipids, indicating strong consistency with the evolution of aroma compounds and lipids during cold plasma processing.

#### 3.5.2 Mantel test analysis of amino acids and differential lipids

**Fig. 8:**
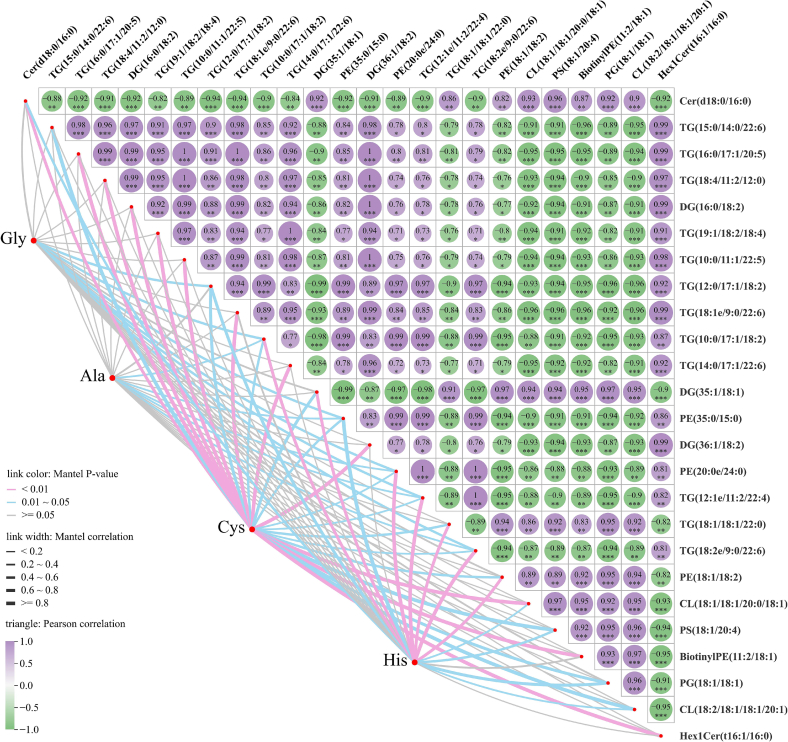
Mantel test analysis of amino acids and differential lipids.

Analysis of amino acids showed that the levels of sweet (glycine and alanine) and bitter (histidine and cysteine) amino acids markedly decreased as cold plasma treatment time increased. Consequently, these four amino acids were selected for Mantel test analysis with the differential lipids, and the results can be found in Fig. 8 and are detailed in Table S8. Mantel test analysis revealed 50 pairs showing significant correlations between differential amino acids and differential lipids. Among them, cysteine exhibited a significant positive correlation with most lipids, particularly DG (36:1/18:2), TG (16:0/17:1/20:5), TG (10:0/11:1/22:5), DG (16:0/18:2), TG (18:4/11:2/12:0), Hex1Cer (t16:1/16:0), TG (18:1e/9:0/22:6), and TG (15:0/14:0/22:6) (*p* < 0.05 and *r* > 0.95). These results suggest that the variations in cysteine levels are closely correlated with the changes in glycerides. Conversely, alanine was negatively correlated with most of the differential lipids. In addition, Mantel test results indicated that the correlations between amino acids and differential lipids were stronger than those between volatile compounds and differential lipids, with smaller *p*-values. This stronger amino acid–lipid correlation implies a closer metabolic or reactional linkage between these metabolites under cold plasma treatment.

## 4 Conclusion

In this study, the effects of the reactive species generated during cold plasma treatment for varying treatment times on the aroma compounds, amino acid composition, and lipid profile of aromatic coconut water were systematically investigated using GC-IMS, amino acid analysis, and lipidomics. The results demonstrated that cold plasma treatment significantly reduced the pH of aromatic coconut water, while the generation of reactive species such as H_2_O_2_, O_2_^−^, NO_2_^−^, and NO_3_^−^ markedly increased with prolonged treatment time. GC-IMS analysis revealed a positive correlation between extended plasma treatment time and the accumulation of octanal, (E)-2-hexenal, and 3-penten-2-one. Amino acid analysis indicated a significant decrease in the content of sweet-tasting amino acids as treatment time increased. Lipidomics identified 25 common differential lipids across the three comparison groups with different treatment times, the majority of which were unsaturated glycerides containing 18:2 and 18:1 fatty-acids. Reactive species generated by cold plasma treatment induce the oxidation and degradation of these unsaturated glycerides, thereby contributing to increased contents of aroma compounds such as octanal. This study provides a theoretical basis for a deeper understanding of the generation and transformation pathways of aroma compounds in aromatic coconut water under cold plasma treatment.

## CRediT authorship contribution statement

**Tao Wang:** Writing – review & editing, Writing – original draft, Methodology, Data curation. **Lilan Xu:** Visualization, Data curation. **Lipin Chen:** Resources, Conceptualization. **Meizhen Xie:** Resources, Conceptualization. **Jiamei Wang:** Resources, Conceptualization. **Weimin Zhang:** Resources, Conceptualization. **Wenxue Chen:** Resources, Funding acquisition. **Yong-Huan Yun:** Writing – review & editing, Resources, Project administration, Funding acquisition.

## Declaration of competing interest

The authors declare that they have no known competing financial interests or personal relationships that could have appeared to influence the work reported in this paper.

## Data Availability

Data will be made available on request.
