# Supplementary material for: Effects of cold plasma treatment time on aroma components, amino acids, and lipids in aromatic coconut water
Source: Food Chem X. 2026 May 13;36:103973. doi: 10.1016/j.fochx.2026.103973 (PMC13208089; doi:10.1016/j.fochx.2026.103973)
Supplement: Supplementary file 1 — Supplementary material. [file mmc1.docx]

Supporting Information

**Effects of cold plasma treatment time on aroma components, amino acids, and lipids in aromatic coconut water**

**2.3 The determination of volatile compounds based on GC-IMS**

GC analysis:

A DB-WAX column (30 m × 0.25 mm × 0.25 μm) was used for GC analysis. The temperature was programmed from an initial temperature of 40°C (held for 3 min) to 180°C at a rate of 4°C/min, followed by a 2 min hold at 180°C. The final post-run temperature was set at 210°C.

IMS analysis:

IMS was performed using a tritium (^3^H) ionization source under an electric field strength of 500 V/cm. The drift tube (53 mm) was maintained at 45°C, with high-purity nitrogen as the drift gas at a flow rate of 150.0 mL/min. The data were collected in positive ion mode.

The calibration solution (a mixed standard of C4–C9 ketones) was analyzed to establish calibration curves for retention time (RT) and retention index (RI). The RI values of volatile compounds were subsequently calculated based on their RTs. Qualitative identification of the volatile compounds was performed by comparing the RI and drift time (DT) values with those of reference standards in the GC-IMS database.

**Table S1** The volatile compounds in aromatic coconut water detected by GC-IMS.

| No. | Compound name | CAS | Formula | RI | Rt [sec] | Dt [a.u.] |
| --- | --- | --- | --- | --- | --- | --- |
| Esters |  |  |  |  |  |  |
| 1 | 2-Methylpropyl propionate | 540-42-1 | C_7_H_14_O_2_ | 1106,3 | 533,218 | 128,241 |
| 2 | Hexyl butanoate | 2639-63-6 | C_10_H_20_O_2_ | 1374,6 | 1,280,302 | 149,076 |
| 3 | Butanoic acid ethyl ester | 105-54-4 | C_6_H_12_O_2_ | 1061,9 | 461,321 | 119,927 |
| 4 | Ethyl hexanoate M | 123-66-0 | C_8_H_16_O_2_ | 1215,7 | 762,231 | 134,674 |
|  | Ethyl hexanoate D | 123-66-0 | C_8_H_16_O_2_ | 1211,8 | 752,524 | 181,039 |
| 5 | Hexyl formate | 629-33-4 | C_7_H_14_O_2_ | 1353,4 | 1194,74 | 13,243 |
| 6 | Methyl caprylate M | 111-11-5 | C_9_H_18_O_2_ | 1373,5 | 1275,78 | 142,862 |
|  | Methyl caprylate D | 111-11-5 | C_9_H_18_O_2_ | 1373,8 | 1,276,915 | 192,891 |
| 7 | Ethyl octanoate M | 106-32-1 | C_10_H_20_O_2_ | 1400,3 | 1,392,583 | 147,677 |
|  | Ethyl octanoate D | 106-32-1 | C_10_H_20_O_2_ | 1399,1 | 1,387,182 | 202,397 |
| 8 | Methyl acetate | 79-20-9 | C_3_H_6_O_2_ | 824,6 | 212,582 | 119,273 |
| 9 | Ethyl acetate M | 141-78-6 | C_4_H_8_O_2_ | 889,1 | 262,451 | 109,478 |
|  | Ethyl acetate D | 141-78-6 | C_4_H_8_O_2_ | 894,2 | 266,791 | 133,989 |
| 10 | Sec-butyl acetate | 105-46-4 | C_6_H_12_O_2_ | 987,2 | 361,457 | 122,718 |
| 11 | Ethyl 3-methylbutanoate | 108-64-5 | C_7_H_14_O_2_ | 1071,5 | 475,961 | 126,617 |
| 12 | Isobutyl 3-methylbutyrate | 589-59-3 | C_9_H_18_O_2_ | 1182,6 | 684,111 | 13,852 |
| 13 | Isopropyl butanoate | 638-11-9 | C_7_H_14_O_2_ | 1012,8 | 392,946 | 168,791 |
| Alcohols |  |  |  |  |  |  |
| 14 | 1-Octen-3-ol | 3391-86-4 | C_8_H_16_O | 1434,3 | 1,555,819 | 114,649 |
| 15 | (E)-2-Hexen-1-ol | 928-95-0 | C_6_H_12_O | 1183,1 | 685,326 | 118,016 |
| 16 | Linalol | 78-70-6 | C_10_H_18_O | 1516,9 | 2,037,486 | 125,577 |
| 17 | 2-Methyl-1-propanol M | 78-83-1 | C_4_H_10_O | 1129,4 | 575,009 | 117,321 |
|  | 2-Methyl-1-propanol D | 78-83-1 | C_4_H_10_O | 1129,7 | 575,557 | 137,604 |
| 18 | 3-Methylbutan-1-ol M | 123-51-3 | C_5_H_12_O | 1242,5 | 831,79 | 124,756 |
|  | 3-Methylbutan-1-ol D | 123-51-3 | C_5_H_12_O | 1242,9 | 832,971 | 148,657 |
| Aldehydes |  |  |  |  |  |  |
| 19 | 2,4-Heptadienal | 5910-85-0 | C_7_H_10_O | 1480,6 | 1,809,566 | 119,218 |
| 20 | (E)-2-Pentenal | 1576-87-0 | C_5_H_8_O | 1076,2 | 483,4 | 111,412 |
| 21 | (E)-2-Hexenal | 6728-26-3 | C_6_H_10_O | 1243,4 | 834,45 | 120,335 |
| 22 | Furfural | 98-01-1 | C_5_H_4_O_2_ | 1470,1 | 1,748,751 | 10,901 |
| 23 | Octanal | 124-13-0 | C_8_H_16_O | 1304,0 | 1,016,949 | 141,455 |
| Ketones |  |  |  |  |  |  |
| 24 | 3-Penten-2-one | 625-33-2 | C_5_H_8_O | 1157,0 | 629,369 | 107,363 |
| 25 | 3-Hydroxy-2-butanone | 513-86-0 | C_4_H_8_O_2_ | 1301,4 | 1,008,146 | 13,215 |
| Heterocycles |  |  |  |  |  |  |
| 26 | 2,6-Dimethylpyridine | 108-48-5 | C_7_H_9_N | 1301,5 | 1008,63 | 108,059 |
| 27 | 2-Methylpyridine | 109-06-8 | C_6_H_7_N | 1261,7 | 885,646 | 134,745 |
| 28 | 2-Ethyl-5-methylpyrazine | 13360-64-0 | C_7_H_10_N_2_ | 1382,6 | 1,314,173 | 166,289 |
| 29 | 2-Acetyl-1-pyrroline | 85213-22-5 | C_6_H_9_NO | 912,8 | 283,486 | 113,573 |
| Others |  |  |  |  |  |  |
| 30 | 2-Butoxyethanol | 111-76-2 | C_6_H_14_O_2_ | 1431,8 | 1,543,329 | 121,517 |
| 31 | Unknown | - | - | 825,7 | 213,363 | 111,249 |

**Table S2** Comparison of amino acid content in coconut water samples / (mg/100 mL).

| Amino acids | Taste profile | Treatment time | | |
| --- | --- | --- | --- | --- |
|  |  | CP_0s | CP_88s | CP_176s |
| Aspartic acid | Umami | 14.93±1.21^a^ | 13.68±0.50^a^ | 13.28±0.30^a^ |
| Threonine* | Sweetness | 5.56±0.42^a^ | 5.12±0.20^a^ | 4.95±0.15^a^ |
| Serine | Sweetness | 11.46±0.88^a^ | 10.69±0.43^a^ | 10.28±0.36^a^ |
| Glutamic acid | Umami | 34.11±2.78^a^ | 32.54±0.69^a^ | 32.72±1.01^a^ |
| Glycine | Sweetness | 5.31±0.29^a^ | 4.74±0.07^b^ | 4.75±0.22^b^ |
| Alanine | Sweetness | 35.86±2.19^a^ | 31.59±0.99^b^ | 33.02±1.23^b^ |
| Cysteine | Bitterness | 3.54±0.44^a^ | 3.02±0.47^b^ | ND |
| Valine* | Bitterness | 9.18±0.61^a^ | 7.89±0.60^a^ | 8.11±0.83^a^ |
| Isoleucine* | Bitterness | 4.27±0.34^a^ | 3.90±0.14^a^ | 4.05±0.32^a^ |
| Leucine* | Bitterness | 7.00±0.49^a^ | 6.45±0.17^a^ | 6.44±0.25^a^ |
| Phenylalanine* | Bitterness | 3.64±0.27^a^ | 3.26±0.06^a^ | 3.35±0.16^a^ |
| Lysine* | Sweetness | 7.76±0.63^a^ | 7.12±0.25^a^ | 6.93±0.15^a^ |
| Histidine | Bitterness | 2.39±0.27^a^ | ND | ND |
| Arginine | Bitterness | 19.55±2.28^a^ | 18.58±0.49^a^ | 19.15±0.30^a^ |
| Proline | Sweetness | 10.53±0.99^a^ | 10.35±0.39^a^ | 10.11±0.59^a^ |
| Essential amino acids | - | 37.41±2.83^a^ | 33.74±0.31^a^ | 33.84±1.82^a^ |
| Total free amino acids | - | 175.09±13.24^a^ | 158.93±3.12^b^ | 157.16±3.20^b^ |

Note: “*” denotes essential amino acids. “ND” indicates not detected. The different letters within the same row represent statistically significant differences (*p* < 0.05).

**Table S3** The top 30 differential lipids between 0 s and 88 s of cold plasma treatment.

|  | Name | *p* | FC | Variable importance in projection (VIP) | False discovery rate (FDR) |
| --- | --- | --- | --- | --- | --- |
| 1 | MG(18:3e) | 0.0001 | 1.0234 | 1.5487 | 0.0635 |
| 2 | TG(8:0/12:0/18:1) | 0.0013 | 1.0066 | 1.3284 | 0.6860 |
| 3 | AcHexCmE(16:0) | 0.0014 | 1.0116 | 1.5334 | 0.1110 |
| 4 | Cer(d20:0/16:0) | 0.0021 | 1.0075 | 1.0418 | 0.5141 |
| 5 | Cer(d12:0/15:0) | 0.0023 | 1.0214 | 1.5045 | 0.3191 |
| 6 | Hex1Cer(t16:1/16:0) | 0.0023 | 0.9492 | 1.5653 | 0.0135 |
| 7 | TG(18:1e/9:0/22:6) | 0.0029 | 0.9403 | 1.5703 | 0.0135 |
| 8 | TG(16:1/11:2/12:0) | 0.0036 | 0.9674 | 1.5049 | 0.1328 |
| 9 | CL(18:2/18:1/18:1/20:1) | 0.0045 | 1.0075 | 1.5111 | 0.0368 |
| 10 | TG(18:1/11:1/14:0) | 0.0051 | 1.0128 | 1.2897 | 0.6807 |
| 11 | TG(18:1/14:0/18:2) | 0.0051 | 1.0071 | 1.2118 | 0.7014 |
| 12 | BiotinylPE(11:2/18:1) | 0.0073 | 1.0342 | 1.4648 | 0.9018 |
| 13 | TG(18:2e/9:0/22:6) | 0.0078 | 0.9425 | 1.5638 | 0.0363 |
| 14 | PS(18:1/20:4) | 0.0101 | 1.0099 | 1.5004 | 0.2003 |
| 15 | TG(12:1e/11:2/22:4) | 0.0103 | 0.9461 | 1.5576 | 0.0363 |
| 16 | TG(14:0/14:0/14:0) | 0.0112 | 1.0310 | 1.0896 | 0.8550 |
| 17 | DG(35:1/18:1) | 0.0117 | 1.0130 | 1.5269 | 0.3504 |
| 18 | Hex1Cer(t16:0/22:4) | 0.0118 | 1.0089 | 1.3203 | 0.3624 |
| 19 | PE(18:1/18:2) | 0.0137 | 1.0085 | 1.4790 | 0.1082 |
| 20 | TG(10:0/18:1/18:2) | 0.0146 | 1.0113 | 1.5404 | 0.1440 |
| 21 | TG(15:0/14:0/22:6) | 0.0152 | 0.9743 | 1.5186 | 0.1259 |
| 22 | LPE(23:0) | 0.0153 | 0.9928 | 1.3546 | 0.3660 |
| 23 | PE(20:0e/24:0) | 0.0184 | 0.9397 | 1.5437 | 0.0417 |
| 24 | TG(18:3e/11:1/22:4) | 0.0186 | 0.9427 | 1.5023 | 0.1082 |
| 25 | TG(16:0/17:1/18:2) | 0.0207 | 0.9698 | 1.4354 | 0.2542 |
| 26 | LPE(24:0) | 0.0211 | 0.9919 | 1.4967 | 0.1085 |
| 27 | Hex1Cer(d18:1/20:4) | 0.0213 | 1.0087 | 1.3069 | 0.4152 |
| 28 | TG(18:1/18:1/22:0) | 0.0227 | 1.0115 | 1.2657 | 0.4372 |
| 29 | TG(12:0/17:1/18:2) | 0.0229 | 0.9681 | 1.5386 | 0.0635 |
| 30 | TG(14:0/17:1/22:6) | 0.0236 | 0.9816 | 1.4934 | 0.1608 |

**Table S4** The top 30 differential lipids between 0 s and 176 s of cold plasma treatment.

|  | Name | *p* | FC | Variable importance in projection (VIP) | False discovery rate (FDR) |
| --- | --- | --- | --- | --- | --- |
| 1 | DG(18:3e/18:1) | 0.0001 | 1.0186 | 1.3270 | 0.2569 |
| 2 | DG(18:1/18:2) | 0.0002 | 0.8563 | 1.3856 | 0.0109 |
| 3 | TG(18:1e/9:0/22:6) | 0.0002 | 0.7099 | 1.3860 | 0.0001 |
| 4 | PE(10:0e/11:2) | 0.0002 | 1.1588 | 1.3852 | 0.0068 |
| 5 | TG(8:0/12:0/18:2) | 0.0003 | 1.0120 | 1.2157 | 0.4797 |
| 6 | DG(36:1/18:2) | 0.0003 | 0.8005 | 1.3812 | 0.0010 |
| 7 | TG(18:2/17:1/22:6) | 0.0004 | 0.8686 | 1.3759 | 0.0031 |
| 8 | PE(15:0/22:4) | 0.0004 | 0.7141 | 1.3860 | 0.0007 |
| 9 | TG(18:1/17:1/22:6) | 0.0007 | 0.8780 | 1.3753 | 0.0057 |
| 10 | TG(16:0/17:1/20:5) | 0.0008 | 0.8116 | 1.3828 | 0.0017 |
| 11 | SM(d18:0/20:2) | 0.0009 | 0.7080 | 1.3838 | 0.0032 |
| 12 | TG(18:4/11:2/18:1) | 0.0010 | 0.7862 | 1.3791 | 0.0009 |
| 13 | PS(18:1/20:4) | 0.0010 | 1.0263 | 1.3638 | 0.0342 |
| 14 | DG(8:0/18:2) | 0.0017 | 0.7507 | 1.3823 | 0.0011 |
| 15 | DLCL(22:0/18:1) | 0.0017 | 1.0280 | 1.3705 | 0.0847 |
| 16 | TG(16:0/18:1/18:1) | 0.0018 | 1.0146 | 1.3543 | 0.3330 |
| 17 | TG(12:0/17:1/22:6) | 0.0018 | 0.8290 | 1.3809 | 0.0011 |
| 18 | TG(18:1/18:1/18:1) | 0.0019 | 1.0193 | 1.2929 | 0.2748 |
| 19 | TG(14:0/13:0/22:6) | 0.0019 | 0.7212 | 1.3748 | 0.0011 |
| 20 | TG(6:0/11:3/22:3) | 0.0021 | 0.7399 | 1.3834 | 0.0003 |
| 21 | DG(16:0/18:2) | 0.0021 | 0.7190 | 1.3716 | 0.0031 |
| 22 | DG(16:1e/16:0) | 0.0026 | 1.0131 | 1.1436 | 0.3396 |
| 23 | DG(35:1/18:1) | 0.0026 | 1.0245 | 1.3777 | 0.0955 |
| 24 | TG(12:0/13:0/22:6) | 0.0028 | 0.7016 | 1.3834 | 0.0103 |
| 25 | Co(Q10) | 0.0028 | 1.0333 | 1.1267 | 0.6670 |
| 26 | Hex1Cer(t16:1/16:0) | 0.0029 | 0.6704 | 1.3795 | 0.0012 |
| 27 | AEA(18:1) | 0.0031 | 1.0278 | 1.2705 | 0.0847 |
| 28 | TG(10:0/11:1/22:5) | 0.0032 | 0.6841 | 1.3692 | 0.0002 |
| 29 | DG(16:1e/18:2) | 0.0035 | 1.0234 | 1.3227 | 0.2329 |
| 30 | TG(6:0/17:1/22:6) | 0.0035 | 0.7481 | 1.3712 | 0.0018 |

**Table S5** The top 30 differential lipids between 88 s and 176 s of cold plasma treatment.

|  | Name | *p* | FC | Variable importance in projection (VIP) | False discovery rate (FDR) |
| --- | --- | --- | --- | --- | --- |
| 1 | PE(15:0/22:4) | 2.35E-05 | 0.7286 | 1.6250 | 0.0005 |
| 2 | TG(6:0/12:0/12:0) | 3.01E-05 | 0.8965 | 1.6164 | 0.0009 |
| 3 | TG(18:1e/9:0/22:6) | 0.0001 | 0.7550 | 1.6251 | 1.07E-05 |
| 4 | TG(16:0/17:1/20:5) | 0.0002 | 0.8331 | 1.6243 | 0.0020 |
| 5 | SM(d18:0/20:2) | 0.0003 | 0.7254 | 1.6238 | 0.0052 |
| 6 | PE(10:0e/11:2) | 0.0007 | 1.1554 | 1.6251 | 0.0081 |
| 7 | TG(12:0/13:0/22:6) | 0.0008 | 0.7154 | 1.6189 | 0.0167 |
| 8 | DG(18:1/18:2) | 0.0008 | 0.8685 | 1.6253 | 0.0167 |
| 9 | DG(35:1/18:1) | 0.0009 | 1.0113 | 1.5442 | 0.2571 |
| 10 | DG(36:1/18:2) | 0.0011 | 0.8155 | 1.6218 | 0.0007 |
| 11 | TG(6:0/17:1/22:6) | 0.0013 | 0.7597 | 1.6118 | 0.0023 |
| 12 | DG(8:0/18:2) | 0.0013 | 0.7623 | 1.6228 | 0.0002 |
| 13 | TG(6:0/11:3/22:3) | 0.0013 | 0.7556 | 1.6215 | 1.07E-05 |
| 14 | Hex1Cer(t18:1/21:1) | 0.0017 | 1.0086 | 1.6158 | 0.8415 |
| 15 | PS(18:1/20:4) | 0.0019 | 1.0163 | 1.5759 | 0.0814 |
| 16 | PG(18:1/18:1) | 0.0024 | 1.0098 | 1.5881 | 0.1270 |
| 17 | TG(15:0/16:0/16:0) | 0.0024 | 1.0049 | 1.5240 | 0.0189 |
| 18 | TG(18:4/11:2/18:1) | 0.0026 | 0.8012 | 1.6154 | 0.0006 |
| 19 | TG(10:0/11:1/22:5) | 0.0026 | 0.6997 | 1.6089 | 1.07E-05 |
| 20 | Hex1Cer(m21:0/18:2) | 0.0030 | 0.9949 | 1.6032 | 0.0341 |
| 21 | Hex1Cer(t16:1/16:0) | 0.0034 | 0.7062 | 1.6098 | 0.0023 |
| 22 | DG(16:0/18:2) | 0.0040 | 0.7381 | 1.6089 | 0.0052 |
| 23 | TG(14:0/13:0/22:6) | 0.0049 | 0.7431 | 1.6121 | 0.0020 |
| 24 | CL(18:2/18:0/16:1/16:1) | 0.0050 | 0.9690 | 1.3559 | 0.8817 |
| 25 | Cer(d18:0/16:0) | 0.0051 | 1.0219 | 1.4963 | 0.0269 |
| 26 | TG(18:4/11:2/12:0) | 0.0055 | 0.7208 | 1.5983 | 0.0027 |
| 27 | TG(18:4/11:2/16:0) | 0.0059 | 0.7233 | 1.6031 | 0.0052 |
| 28 | TG(18:1/17:1/22:6) | 0.0068 | 0.8893 | 1.6130 | 0.0090 |
| 29 | TG(19:1/18:1/18:4) | 0.0071 | 0.8280 | 1.5972 | 0.0052 |
| 30 | TG(12:0/17:1/22:6) | 0.0082 | 0.8565 | 1.5876 | 0.0149 |

**Table S6** The correlation results of top 30 positive pairs among differential lipids.

| Lipid 1 | Lipid 2 | r | *p* |
| --- | --- | --- | --- |
| PE (20:0e/24:0) | TG (12:1e/11:2/22:4) | 0.9992 | 5.60E-11 |
| TG (12:1e/11:2/22:4) | TG (18:2e/9:0/22:6) | 0.9990 | 9.75E-11 |
| PE (20:0e/24:0) | TG (18:2e/9:0/22:6) | 0.9983 | 7.26E-10 |
| TG (19:1/18:2/18:4) | TG (14:0/17:1/22:6) | 0.9979 | 1.40E-09 |
| TG (16:0/17:1/20:5) | DG (36:1/18:2) | 0.9976 | 2.25E-09 |
| DG (16:0/18:2) | DG (36:1/18:2) | 0.9969 | 5.33E-09 |
| TG (16:0/17:1/20:5) | TG (18:1e/9:0/22:6) | 0.9966 | 7.66E-09 |
| TG (10:0/11:1/22:5) | DG (36:1/18:2) | 0.9964 | 9.40E-09 |
| TG (18:4/11:2/12:0) | DG (36:1/18:2) | 0.9964 | 9.61E-09 |
| TG (18:4/11:2/12:0) | TG (10:0/11:1/22:5) | 0.9958 | 1.59E-08 |
| TG (16:0/17:1/20:5) | TG (10:0/11:1/22:5) | 0.9952 | 2.57E-08 |
| TG (18:1e/9:0/22:6) | Hex1Cer (t16:1/16:0) | 0.9948 | 3.23E-08 |
| TG (18:4/11:2/12:0) | DG (16:0/18:2) | 0.9936 | 6.72E-08 |
| TG (15:0/14:0/22:6) | Hex1Cer (t16:1/16:0) | 0.9932 | 8.39E-08 |
| PE (35:0/15:0) | TG (12:1e/11:2/22:4) | 0.9930 | 9.26E-08 |
| PE (35:0/15:0) | PE (20:0e/24:0) | 0.9923 | 1.29E-07 |
| TG (16:0/17:1/20:5) | DG (16:0/18:2) | 0.9923 | 1.29E-07 |
| TG (16:0/17:1/20:5) | Hex1Cer (t16:1/16:0) | 0.9923 | 1.32E-07 |
| TG (10:0/17:1/18:2) | PE (35:0/15:0) | 0.9920 | 1.49E-07 |
| TG (18:1e/9:0/22:6) | DG (36:1/18:2) | 0.9912 | 2.12E-07 |
| TG (16:0/17:1/20:5) | TG (18:4/11:2/12:0) | 0.9909 | 2.34E-07 |
| TG (10:0/17:1/18:2) | TG (12:1e/11:2/22:4) | 0.9904 | 2.80E-07 |
| PE (35:0/15:0) | TG (18:2e/9:0/22:6) | 0.9901 | 3.11E-07 |
| TG (10:0/17:1/18:2) | PE (20:0e/24:0) | 0.9897 | 3.62E-07 |
| TG (12:0/17:1/18:2) | PE (35:0/15:0) | 0.9893 | 4.07E-07 |
| DG (36:1/18:2) | Hex1Cer (t16:1/16:0) | 0.9886 | 5.10E-07 |
| DG (16:0/18:2) | TG (10:0/11:1/22:5) | 0.9880 | 6.08E-07 |
| DG (16:0/18:2) | Hex1Cer (t16:1/16:0) | 0.9874 | 7.23E-07 |
| DG (16:0/18:2) | TG (18:1e/9:0/22:6) | 0.9863 | 9.90E-07 |
| TG (10:0/17:1/18:2) | TG (18:2e/9:0/22:6) | 0.9857 | 1.14E-06 |

**Table S7** The Mantel test results of volatile compounds and differential lipids.

| Volatile compounds | Lipids | r | *p* |
| --- | --- | --- | --- |
| (E)-2-Hexenal | TG (18:2e/9:0/22:6) | 0.8663 | 0.0040 |
| 3-Penten-2-one | TG (18:2e/9:0/22:6) | 0.8475 | 0.0070 |
| (E)-2-Hexenal | PE (20:0e/24:0) | 0.8450 | 0.0040 |
| (E)-2-Hexenal | TG (12:1e/11:2/22:4) | 0.8439 | 0.0120 |
| 3-Penten-2-one | TG (12:1e/11:2/22:4) | 0.8263 | 0.0150 |
| 3-Penten-2-one | PE (20:0e/24:0) | 0.8261 | 0.0070 |
| (E)-2-Hexenal | PE (35:0/15:0) | 0.7672 | 0.0120 |
| 3-Penten-2-one | PE (35:0/15:0) | 0.7376 | 0.0140 |
| (E)-2-Hexenal | TG (10:0/17:1/18:2) | 0.7092 | 0.0080 |
| 3-Penten-2-one | TG (10:0/17:1/18:2) | 0.7039 | 0.0070 |
| (E)-2-Hexenal | DG (35:1/18:1) | 0.6945 | 0.0010 |
| (E)-2-Hexenal | PE (18:1/18:2) | 0.6935 | 0.0050 |
| 3-Penten-2-one | DG (35:1/18:1) | 0.6551 | 0.0090 |
| Octanal | TG (18:2e/9:0/22:6) | 0.6491 | 0.0080 |
| 3-Penten-2-one | PE (18:1/18:2) | 0.6474 | 0.0050 |
| (E)-2-Hexenal | TG (12:0/17:1/18:2) | 0.6337 | 0.0130 |
| Octanal | TG (12:1e/11:2/22:4) | 0.6292 | 0.0160 |
| Octanal | PE (20:0e/24:0) | 0.6240 | 0.0080 |
| (E)-2-Hexenal | PG (18:1/18:1) | 0.6201 | 0.0060 |
| (E)-2-Hexenal | TG (18:1/18:1/22:0) | 0.6149 | 0.0080 |
| 3-Penten-2-one | TG (12:0/17:1/18:2) | 0.6099 | 0.0200 |
| 3-Penten-2-one | PG (18:1/18:1) | 0.5900 | 0.0190 |
| Octanal | PE (35:0/15:0) | 0.5412 | 0.0220 |
| 3-Penten-2-one | TG (18:1/18:1/22:0) | 0.5355 | 0.0110 |
| Octanal | TG (10:0/17:1/18:2) | 0.5124 | 0.0160 |
| (E)-2-Hexenal | Cer (d18:0/16:0) | 0.4809 | 0.0130 |
| Octanal | DG (35:1/18:1) | 0.4789 | 0.0240 |
| 3-Penten-2-one | Cer (d18:0/16:0) | 0.4706 | 0.0220 |
| Octanal | PE (18:1/18:2) | 0.4643 | 0.0200 |
| Octanal | TG (12:0/17:1/18:2) | 0.4412 | 0.0340 |
| (E)-2-Hexenal | CL (18:1/18:1/20:0/18:1) | 0.4359 | 0.0150 |
| (E)-2-Hexenal | PS (18:1/20:4) | 0.4267 | 0.0190 |
| Octanal | PG (18:1/18:1) | 0.4208 | 0.0390 |
| 3-Penten-2-one | PS (18:1/20:4) | 0.4017 | 0.0270 |
| (E)-2-Hexenal | CL (18:2/18:1/18:1/20:1) | 0.3795 | 0.0390 |
| 3-Penten-2-one | CL (18:2/18:1/18:1/20:1) | 0.3562 | 0.0420 |
| Octanal | TG (18:1/18:1/22:0) | 0.3530 | 0.0370 |
| 3-Penten-2-one | CL (18:1/18:1/20:0/18:1) | 0.3354 | 0.0390 |

**Table S8** The Mantel test results of amino acids and differential lipids.

| Amino acids | Lipids | r | *p* |
| --- | --- | --- | --- |
| Cys | DG (36:1/18:2) | 0.9884 | 0.004 |
| Cys | TG (16:0/17:1/20:5) | 0.9847 | 0.005 |
| Cys | TG (10:0/11:1/22:5) | 0.9826 | 0.005 |
| Cys | DG (16:0/18:2) | 0.9761 | 0.006 |
| Cys | TG (18:4/11:2/12:0) | 0.9730 | 0.005 |
| Cys | Hex1Cer (t16:1/16:0) | 0.9681 | 0.004 |
| Cys | TG (18:1e/9:0/22:6) | 0.9677 | 0.004 |
| Cys | TG (15:0/14:0/22:6) | 0.9527 | 0.01 |
| His | TG (18:2e/9:0/22:6) | 0.9207 | 0.003 |
| His | PE (20:0e/24:0) | 0.9052 | 0.003 |
| His | TG (12:1e/11:2/22:4) | 0.8994 | 0.005 |
| Cys | TG (14:0/17:1/22:6) | 0.8898 | 0.006 |
| Cys | TG (19:1/18:2/18:4) | 0.8637 | 0.008 |
| Cys | PS (18:1/20:4) | 0.8315 | 0.012 |
| Cys | Biotinyl PE (11:2/18:1) | 0.8282 | 0.006 |
| Cys | CL (18:1/18:1/20:0/18:1) | 0.8228 | 0.007 |
| His | PE (35:0/15:0) | 0.8200 | 0.011 |
| Cys | CL (18:2/18:1/18:1/20:1) | 0.8163 | 0.012 |
